# The legal path for priority setting in Chile: a critical analysis to improve health planning and stewardship

**DOI:** 10.3389/fpubh.2023.1302640

**Published:** 2024-01-08

**Authors:** Manuel Antonio Espinoza, Baltica Cabieses, Carolina Goic, Alejandro Andrade

**Affiliations:** ^1^Departamento de Salud Pública, Pontificia Universidad Catolica de Chile, Santiago, Chile; ^2^Centro para la Prevención y Control del Cancer (CECAN) & Departamento de Hemotaologia y Oncología, Pontificia Universidad Catolica de Chile, Santiago, Chile; ^3^Center for Global Intercultural Health, University for Development, Santiago, Chile; ^4^Chilean Federation of Rare Diseases, Santiago, Chile

**Keywords:** health priorities, Chile, health planning, health legislation, health policy

## Abstract

Health systems have committed their path to universal health coverage using health planning to accomplish their goals of efficiency, equity and sustainability. Chile, a high-income country with a public-private mix health system, has made significant progress through several successive health policies implemented in the last 20 years which have been consistent with this approach. However, in the last 5 years, the national congress has produced several disease-specific laws, which have been mainly promoted by the civil society. These laws indicate the actions the health authority must perform to tackle the needs of the affected population, which ultimately determine the priorities of the health system. We argue that this legal pattern has become an alternative path to priority-setting, as opposed to health planning. We claim this “legal path” is a mechanism used by civil society in a context where the health authority fails to implement a robust and legitimate prioritization process. Although these laws have brought benefits to patients suffering the corresponding conditions, we highlight this approach does not guarantee improvements in equity, efficiency and health system performance. Instead, we advocate for taking back the control of the priority-setting based on health planning, through a new institutionalization of health technology assessment and quality of care.

## Background

Universal health coverage (UHC) is one of the main goals of health systems, but also one the their main challenges. The health authority in every country is forced to prioritize among several investment alternatives given their financial restrictions. Furthermore, the empowered civil society demands greater accountability for the decisions of the health system, which must respond to the unmet needs of the population and be consistent with its objectives such as efficiency and equity. In this context, health systems need to strengthen their capacities in health planning, evidence appraisal, and social valuation of new prospects, to make the most legitimate and fair resource allocation possible.

The Chilean health system is organized as a private-public mix of payers (insurers) and providers under the Ministry of Health’s (MoH) stewardship (1). In 2005 the Chilean health system had its most important reform in the last 40 years. The Health Benefits plan (HBP) “Explicit Health Guarantees (EHG)” was one of the most significant changes (2). This HBP was organized through a list of prioritized health problems or diseases, which provides guarantees of access, financial protection, quality of provision and timely delivery of services according to health needs. The EHG established a health planning logic in the health system, allowing the health authority to govern actions for constant progress to UHC. Unfortunately, budgetary restrictions on EHG have hindered its growth, leaving patients with and without prioritized health conditions with many unmet needs (3).

Ten years later, in 2015 Chile launched another complementary universal HBP, focusing on high-cost technologies: the “System of Financial Protection of High-Cost: a tribute to Mr. Ricarte Soto” (4). The so-called “Ricarte Soto Law” (RSL) owes its name to the leader of the social movement “The March of the Patients,” which demanded more access to high-cost health technologies in the health system. Alongside the RSL, the MoH set up a Health Technology Assessment (HTA) department, which leads a HTA process that brought improvements in transparency and accountability compared to previous priority-setting exercises. This new HTA process strengthened the health planning capacities of the health authority but still needs significant improvements in content and procedures (5).

The health expenditure has grown significantly in Chile in the last decade (6), achieving the average percentage of the gross domestic product of the OCDE countries in 2022 (9%) (7). However, its public expenditure is still below the average (5.6%), with a large out-of-pocket expenditure, which reaches 29.8%, 11.7% higher that the OCDE average (18.1%). Compared to other Latinamerican countries, Chile shows the best index in access and quality of care (78), followed by Cuba (76) and Costa Rica (74). However, it depicts an index of coverage to essential health services of 70, in the 15th position of a table led by Cuba (83) and Uruguay (80) (8).

Despite all these efforts, there is a general perception of low protection against catastrophic diseases in the Chilean population. Furthermore, the Chilean civil society, especially patients’ organizations and their representatives, have gained knowledge and capacity to express their demands to the health authority (9). Also, politicians have claimed they will work on a more profound health reform to sort out all remaining challenges, particularly around unequal access to healthcare. Nevertheless, legislative discussion about the reform has been postponed for almost 20 years. In this manuscript, we present an analysis of this new social scenario. We argue that Chile has left behind the health planning for priority setting, which has been replaced by what we present here as the “legal path for priority setting.”

## The first step of the legal path: the cancer law

Although both EHG and RSL included services and technologies to provide timely access to diagnostics and treatments for cancer, civil society claimed that cancer care was insufficient for the magnitude of the problem. Cancer represents a high burden of disease and expenditure in Chile (10), becoming the first cause of death in the country in 2019 (11). Furthermore, the supply side is concentrated in highly populated urban cities, partially explaining large geographic health inequalities in mortality (12). The lack of response from the health authority to the growing perception of social injustice in the community led civil society to demand a specific Cancer law, which was finally launched in 2020 (13).

The cancer law draws how the health authority should run the policies in this matter, indicating actions and responsibilities to plan: (i) the capacity development along the country (e.g., human resources and infrastructure); (ii) to incorporate civil society in these policies; (iii) to create a national cancer registry; and (iv) to ensure research and innovation to improve population health outcomes and the health system performance. The historic timeline that ended in this law is presented in Table 1.

**Table 1:** Milestones of the cancer law 21258.

Milestone	Develop
15.01.2013 National Cancer Forum creation leaded by Dr. Jorge Jimenez de la Jara	To build a participatory national strategy to address cancer, civil society, scientific societies, patient organizations, researchers, clinicians, and different actors related to cancer elaborated proposals into working groups in: Prevention, Research, Communication, and Public-Private Alliances.
24.01.2014 National Cancer Forum presents the citizen bases of the cancer bill to senators and deputies	The proposal prepared by the Forum is delivered to the minister of health and senators and deputies so that it can be processed in Congress. The proposal considers prevention, early detection, treatment and research in cancer, public-private collaboration, the registry, and the cancer plan.
04.11.2014 Presentation of the National Cancer Law Project	The national cancer bill was presented by a group of Senators led by Senator Carolina Goic with the unanimous support of all sitting senators and deputies. For its discussion, the text required the support of the President and the authorization of the resources involved by the Ministry of Finance.
18.11.2018 Citizen march for the national cancer law	Patient and civil society organizations promoted the cancer bill to be placed on the public agenda and to commit the president of the republic to send it to the parliament with his support.
10.12.2018 Presentation of the National Cancer Bill by the President Presentation of the first national cancer plan for Chile	Formal presentation of the bill that establishes a national cancer law by President Sebastián Piñera. The legislative processing of the Project began in the Senate Health Commission. The bill included the obligation of the government to elaborate the national cancer plan and its update every 5 years, the creation of the National Cancer Commission, the national cancer registry, the oncology network throughout the country, the national cancer fund and tax exemptions for cancer donations
20.12.2018–06.05.2019 Discussion of the bill with audiences of civil society organizations	The discussion of the project in the Congress considered exhibitions and public hearings from foundations, medical societies, health professionals, representatives of public institutions, and other civil society organizations. All contributed with their perspectives, which were considered in the legal text approved in the Senate.
3.2019–4.2019 Citizen participation process through Cancer Colab and presentation of proposals for the bill text.	The national cancer law is the first in the Chilean Congress that allowed citizen participation in the drafting of the text, through a participatory process through artificial intelligence tools carried out with the support of the Collective Intelligence Center of the Massachusetts Institute of Technology. As a result, the senators presented 24 proposals for modifications to the text that collected observations from civil society, including labor protection for people with cancer and better mechanisms for reviewing the national plan.
5 October 2020—approval of the law by the Congress of the Republic	The law was approved with broad consensus in the congress. After its unanimous approval in the Senate and the Chamber of Deputies, the cancer law completed its processing in Congress with broad political and citizen support.
26.08.2020 Promulgation of the National Cancer Law	Publication in the official journal of Law 21258 that creates the national cancer law, which pays posthumous tribute to Dr. Claudio Mora.

The law also addresses current barriers in the delivery of cancer services, which include the lack of specialists, capacities for diagnostic, surgery, radiotherapy and medical treatments. For example, it mandates the elaboration of a national cancer plan, which defines specific actions for the next 5 years to improve cancer services (14). It creates the national oncology network categorized in low, medium and high complexity. It behests the MoH to elaborate clinical guidelines for all cancers. Finally, it entitles the right to diagnostic confirmation and to receive treatment, which forces the MoH to implement access mechanisms to healthcare.

Although the law only provided some additional funds (USD 23,000 MM annually approximately) to improve infrastructure, equipments and registry; it highlighted the problem in the public agenda facilitating the creation of the high-cost drugs fund for cancer (*Drogas de alto costo* in Spanish, DAC), which was included in the annual national budget law of the country. This fund was allocated to provide medicines that were not covered yet by EHG or RSL, to beneficiaries of the national public insurer. Because this initiative was not part of the law, the priority setting and allocation process did not follow a structured HTA. However, a deliberative exercise among experts convened for this purpose by the MoH took place.

## The following steps: the law as a means for non-HTA led priority setting processes

The pandemic obliged the Chilean health system to focus its resources on the urgent needs of COVID-19, leaving behind many diagnostics and treatments. Yet, the health system has claimed not to have additional resources, such as expanding coverage to new treatments for many health conditions. Furthermore, the health reform announced by the current government seemed to be postponed once again. In this context, where there are no concrete actions to improve access to healthcare in the short term, it seemed that the health agenda could only be pushed forward through the development of new laws, as in the recent case of the Cancer law.

Likewise, we have observed similar legal initiatives after the cancer law in the last few years. First, the law that recognizes and protects the rights to mental health services was launched in May 2021 (15). Second, the law that entitles palliative care and protects the rights of people who suffer terminal or severe diseases was published in October 2021 (16). Third, the law for fibromyalgia and non-oncologic chronic pain saw the light in February 2023 (17). Finally and more recently, the project for a rare diseases law is under discussion in the national congress. Table 2 shows a summary of the laws related to priority setting operating in Chile.

**Table 2:** Laws enacted in Chile regulating priority setting in healthcare.

Law	Year	Priority setting mechanism	Main prioritization criteria
Law 21531 Fibromyalgia and non-oncologic chronic pain	2023	Mechanism not defined in the law	Criteria not defined in the law
Law 21430, Protection of children and adolescents’ rights	2022	Prioritization of access to public health services for children and adolescents whose rights have not been met.	Children in need
Law 21375 Right to palliative care	2021	Mechanism not defined in the law	Criteria not defined in the law
Law 21331 Acknowledges and protects the right of people to have access to mental health services	2021	Mechanism not defined in the law	Services defined based on best evidence available and cost-effectiveness.
Law 20850, access to high cost technologies	2015	Health technology assessment process	Effectiveness, safety, efficiency, affordability, feasibility for implementation, social values
Law 20584 On the rights and duties of patients regarding actions related to healthcare	2012	It establishes criteria for preferent access to health care	People older than 60 years old, people with disabilities, and their careers.
Law 19966 Explicit Health Guarantees National Health Benefit Plan	2004	Prioritization of health conditions and services that will be incorporated into the National Health Benefit Plan	Burden of disease, Probability of financial catastrophe, Social preferences, cost-effectiveness
Ley 19650, about healthcare access in emergency services	2002	Patients in need of emergency services must receive healthcare, eliminating all financial barriers.	Need of emergency care
Law 19451, on human organ donations and transplants	1995	Prioritization based on the severity of the patient’s condition	Severity of the patient’s condition

All these initiatives do not bring additional specific funds to provide new services or technologies. However, it forces the MoH to take action to improve access to care or improve patients’ quality of life. Ultimately, the MoH needs to allocate resources to these health conditions to accomplish the law. Therefore, these initiatives have an impact on prioritizing these conditions over others. In other words, instead of a formal HTA-led priority setting for health planning in Chile, this process is being driven by individual disease-specific laws.

To the best of our knowledge, it is the first time Chile has produced so many legal initiatives that address single health problems or diseases in such a short period. We argue they express an alternative path to a formal priority-setting; that is, “the legal path,” which has shown in the last few years to be more efficacious than health planning, for example, through EHG or RSL. These initiatives showed to be in tune with the population demands and the political will in Congress. Moreover, they have received more support from the health authority than other actions to strengthen health planning, for example, the Health Technology Assessment (HTA) institutionalization.

Furthermore, we identified some advantages of the legal path for priority setting (Table 3). First, because the legal path has occurred as a consequence of the actions of the civil society, it provides a signal of the high degree of empowerment and capacity to impact policy making. Second, the process in the congress to produce a law involves the consideration of all stakeholders’ views, receiving indications of several shares and the commitment of the health authority and the government. Given the level of participation and the deepness achieved along the discussion, the content in the law might gain legitimacy (18). Third, civil society representatives gain deep understanding of the making-law process, which helps adjusting expectations about the policy.

**Table 3:** Advantages and disadvantages of the legal path to priority setting.

Advantages	Justification
Empowerment of the civil society	When the legal path is in place, civil society has demonstrated they can position a topic in the legal agenda, commit members of the parliament, and influence the public opinion. They became one valid stakeholder for health policy development.
The discussion in the parliament improves the legitimacy of the content in the law, which often guides the policy implementation.	The process in the congress to produce a law involves the consideration of all stakeholders’ views, receiving indications of several shares and the commitment of the health authority and the government. Given the level of participation and the deepness achieved along the discussion, the content in the law gains legitimacy.
Adjustments of expectations related to the policy	Civil society representatives who participate in the discussion of the law-making process can better understand certain constraints, which help adjust their expectations regarding the policy.
Disadvantages	Justification
The legal path does not consider the opportunity cost of an alternative resource allocation.	Disease-specific laws amplifies the relevance of one health problem over the rest. They may force-directly or indirectly-to health authorities to derive resources to the problem, with no consideration of the benefits forgone elsewhere in the healthcare system.
The legal path may not favor equity in access to healthcare.	The problem entitled in the law is often positioned by organized groups with relative greater advocacy capacities than others. Hence, putting some diseases ahead implicitly means leaving less advantaged patients or citizens behind. They might be groups with no support or resources to advocate for their health problems.
The legal path weakens the governing role of the health authority.	If many legal indications emerge from the parliament obliging the health authority to take actions on public health matters, the governance of the health sector is at risk. Both, public health, and health care management require political and technical skills that must remain in the government bodies responsible for the stewardship of the health system.

On the other hand, we acknowledge some disadvantages or risks of using the legal path to priority setting. First, a legal-based priority setting fails in considering the opportunity cost of an alternative allocation of resources. In a recent study in Colombia, it was estimated that providing funding to 10 new high-cost drugs produces a net health loss of 88,000 Quality Adjusted Life Years (19). If health planning through HTA is left behind, there is no possibility to account for these losses. Second, a disease-specific law favors patients suffering that specific disease in detriment to other patients for whom there is no specific law. Further, it can also produce inequities in individuals who are beneficiaries of the same law; especially when it does not provide the instruments and resources to obey the law. For example, the Patient Protection and Affordable Care Act in USA expanded private insurance to young adults. However, it did it only for Asian and white patients in higher income neighborhoods (20). Third, the risk of weakening of the health authority governance, which contrasts with the positive effect of empowering the civil society. While the latter is desirable when is used responsibly, the former is detrimental for the health system because the authority losses control of the actions and priorities that must be undertaken to achieve the global goals of the system (21, 22).

Interestingly, civil society is not only demanding more resources. For example, the law and national plan for rare diseases (NPRD) proposed by patients groups and social organizations, highlights the need for an independent body responsible for HTA and quality of care. The NPRD recognizes the challenges of the health system in financing all medicines for rare diseases and how this may affect its sustainability. Indeed, instead of producing tension with the system, it proposes reasonable actions to move forward including the development of value frameworks, improving capacities for HTA and priority setting standards.

Sooner rather than later, the healthcare system must regain health planning control. Leaving the parliament to define what diseases should receive attention first and which ones should wait, does not provide a guarantee of equitable and efficient results, or better health system performance. Instead, the health authority must lead the priority setting through coherent, systematic and transparent processes that integrate all relevant visions, including clinical, economic, and social perspectives.

## Concluding remarks

The legal path is a democratic response of the civil society supported by the parliament to a weak health planning and decision-making process. We argue that the health authority needs to take back control of health planning through improved coherent, systematic and transparent priority-setting processes. We recommend creating a new independent body in charge of the HTA and the quality of care, such as clinical guidelines, protocols and other corresponding norms. We argue that the new body and its institutional arrangement must provide the support to achieve socially legitimate decisions about priority-setting that affect patients and their families. And ultimately, it is a signal of good health system performance that contributes to restoring trust and reliability in the health authority in the country and beyond.

## Data availability statement

The raw data supporting the conclusions of this article will be made available by the authors, without undue reservation.

## Author contributions

ME: Conceptualization, Funding acquisition, Writing – original draft, Writing – review & editing. BC: Funding acquisition, Writing – review & editing. AA: Writing – review & editing. CG: Writing – review & editing.
